# Reliable identification at the species level of *Brucella *isolates with MALDI-TOF-MS

**DOI:** 10.1186/1471-2180-11-267

**Published:** 2011-12-23

**Authors:** Florigio Lista, Frans AG Reubsaet, Riccardo De Santis, Rene R Parchen, Ad L de Jong, Jasper Kieboom, Anton L van der Laaken, Ingrid AI Voskamp-Visser, Silvia Fillo, Hugo-Jan Jansen, Jan Van der Plas, Armand Paauw

**Affiliations:** 1 Department of Earth, Environmental, and Life Sciences, TNO, Lange Kleiweg 137, Rijswijk, P.O Box 45, The Netherlands; 2 Department of Bacteriology, Diagnostic Laboratory for Infectious Diseases and Perinatal Screening, National Institute of Public Health and the Environment (RIVM), Antonie van Leeuwenhoeklaan 9, Bilthoven 3721, MA, The Netherlands; 3 Histology and Molecular Biology Section, Army Medical and Veterinary Research Center, Via Santo Stefano Rotondo 4, 00184 Rome, Italy; 4 Military Health Care Expertise Co-ordination Centre, Ministry of Defence, Noodweg 37, Hilversum 1213, PW, The Netherlands

## Abstract

**Background:**

The genus *Brucella *contains highly infectious species that are classified as biological threat agents. The timely detection and identification of the microorganism involved is essential for an effective response not only to biological warfare attacks but also to natural outbreaks. Matrix-assisted laser desorption/ionization time-of-flight mass spectrometry (MALDI-TOF-MS) is a rapid method for the analysis of biological samples. The advantages of this method, compared to conventional techniques, are rapidity, cost-effectiveness, accuracy and suitability for the high-throughput identification of bacteria. Discrepancies between taxonomy and genetic relatedness on the species and biovar level complicate the development of detection and identification assays.

**Results:**

In this study, the accurate identification of *Brucella *species using MALDI-TOF-MS was achieved by constructing a *Brucella *reference library based on multilocus variable-number tandem repeat analysis (MLVA) data. By comparing MS-spectra from *Brucella *species against a custom-made MALDI-TOF-MS reference library, MALDI-TOF-MS could be used as a rapid identification method for *Brucella *species. In this way, 99.3% of the 152 isolates tested were identified at the species level, and *B. suis *biovar 1 and 2 were identified at the level of their biovar. This result demonstrates that for *Brucella*, even minimal genomic differences between these serovars translate to specific proteomic differences.

**Conclusions:**

MALDI-TOF-MS can be developed into a fast and reliable identification method for genetically highly related species when potential taxonomic and genetic inconsistencies are taken into consideration during the generation of the reference library.

## Background

The genus *Brucella *contains highly infectious species that have been found to cause infections in a wide variety of mammals. Most *Brucella *species have a narrow host range. Infection in humans arises from direct or indirect contact with infected animals or through consumption of contaminated meat or dairy products [1]. Diagnostic laboratory workers are also at risk; 2% of all cases of brucellosis are laboratory acquired. Person-to-person transmission is extremely rare [1-3]. Characteristically, *Brucella *species have a low infectious dose and are capable of transmission via aerosols, and the treatment of infections is lengthy with a risk of complications. For these reasons, *Brucella *is classified as a potential warfare threat agent, and *Brucella suis *has been weaponized in the past by the United States, the former Soviet Union, and China [4].

*Brucella *species belong to the family *Brucellaceae *in the order *Rhizobiales *of the class *Alphaproteobacteria *and are small, non-motile Gram-negative rods. Until recently, six species, some of which may be subdivided into biovars, were assigned to the *Brucella *genus. These species are *Brucella abortus *(seven biovars), *Brucella melitensis *(three biovars), *Brucella suis *(five biovars), *Brucella ovis*, *Brucella canis*, and *Brucella neotomae*. However, four new species have recently been described. Three of these species were isolated from sea mammals and 'wild' mammals: *Brucella ceti*, *Brucella pinnipedialis*, and *Brucella microti *[5-10]. Finally, a new species, *Brucella inopinata*, was isolated from a breast implant (strain BO1) and from a lung biopsy (strain BO2) [11,12].

The *Brucella *species primarily considered to be pathogenic for humans are *B. melitensis*, *B. suis *(biovars 1, 3, and 4), *B. abortus*, and sporadically *B. canis *[1,2,13]. *B. suis *biovars 2 and 5 are considered not to be human pathogens because no human cases have been documented for these agents [13].

The DNA-DNA hybridization results suggest that the classification system used for *Brucella *is open to debate. Among the different *Brucella *species, the DNA-DNA hybridization relatedness varies from 87% to 99%, indicating that the *Brucella *species may actually be considered a single species [13-15]. However, the traditional nomenclature was maintained because the specific host range and pathogenicity differ among the *Brucella *species [1]. The conventional methods used to identify *Brucella *isolates are complex, labor-intensive, and time consuming. In addition, *Brucella *is a potential health hazard to laboratory personnel. Traditionally, the identification of *Brucella *species is mainly based on host specificity, pathogenicity, and minor phenotypic differences that are determined using several separate tests, which include tests for the oxidation of carbohydrate and amino acid substrates, phage sensitivity, CO_2 _requirement, H_2_S production, serum agglutination, and growth in the presence of thionine and basic fuchsine [1]. The scheme to discriminate to the level of biovars is inconclusive because the biological differences between the biovars described are limited, and the interpretation of the results can be subjective [13]. In addition, some *Brucella *isolates appear unable to be typed [13].

DNA-based approaches have been widely introduced to identify microorganisms, including *Brucella *species. A relatively rapid approach is the 'Bruce-ladder', a multiplex PCR that is able to distinguish the six classical species [13,16]. To complement the 'Bruce-ladder', a single PCR was added to distinguish the marine mammal-derived *Brucellae *as well. This method, called *bp*26 PCR, is based on the IS*711 *[13,16].

Another method, mainly developed for the epidemiological investigation of outbreaks, is multilocus variable-number tandem repeat analysis (MLVA). MLVA is based on the differences in the number of tandem repeats in several loci of the bacterial chromosome [17]. The MLVA developed for *Brucella *has been proven to be a reliable, reproducible, and highly discriminatory method that is able to classify all of the *Brucella *strains [13,18-20]. In this study, we the previously described MLVA-16 assay to identify *Brucella *species was used [13,18-20].

Genomic studies have shown that the nomenclature for several *Brucella *species is not consistent if the genetic relationships among species are considered to be the gold standard for discriminating between species [20]. For example, *B. ceti *is divided into two separate groups, one of which is more closely related to *B. pinnipedialis *than to the other group of *B. ceti *[20]. Additionally, *B. suis *biovar 5 is more related to *B. ceti*, *B. neotomae*, *B. pinnipedialis *and *B. ovis *than to the other *B. suis *biovars [20].

The timely detection and rapid identification of the microorganisms involved are essential for the most-effective response to an infectious disease outbreak, regardless of whether the outbreak is natural or deliberate. This rapid identification is necessary not only to treat patients effectively but also to establish outbreak management, source tracing, and threat analyses.

Matrix-assisted laser desorption/ionization time-of-flight mass spectrometry (MALDI-TOF-MS) is a rapid method used to analyze biological differences in microorganisms. MALDI-TOF-MS emerged as a new diagnostic tool in established microbiological laboratories [21]. The advantages of MALDI-TOF-MS over conventional techniques are that it is a fast, cost-effective, accurate method, which is suitable for the high-throughput identification of bacteria by less-skilled laboratory personnel because preliminary identification steps are unnecessary [21-24]. The bacteria are identified by comparing the obtained MS spectra to the MS spectra or profiles of MS spectra from a reference library. Hence, the reliability of the identification is based on the content and quality of this library, among other factors. Recently, a reference library to identify *Brucella *species was constructed using 12 *Brucella *strains, but using this '*Brucella *library', the discrimination was insufficient for identification at the species level [25]. In contrast, reliable identification at the species level was shown for other genetically closely related species, such as *Fransicella *species, *Bacillus *species, and species from the *Burkholderia cepacia *complex [26-28].

The aim of this study was to improve identification using MALDI-TOF-MS at the species level of *Brucella*. Therefore, a custom reference library was constructed with strains that represent the known genetic variation of *Brucella *at the species and biovar level according to MLVA. Subsequently, this custom reference library was evaluated using 152 *Brucella *isolates that were identified using MLVA.

## Methods

### Bacterial strains

Seventeen of the 170 isolates included in this study are reference strains representing the classical *Brucella *species, and only the classical reference strain for *B. suis *biovar 4 is missing (Additional file 1: Table S1) [1]. The 170 isolates included in the study were all typed using MLVA [19]. The *Brucella *isolates originated from K. Walravens, Veterinary and Agrochemical Research Centre (CODA/CERVA), Operational Direction of Bacterial Diseases, Unit of Pathology, Brussels, Belgium, and from the department of Bacteriology of the National Institute for Public Health and the Environment (RIVM), Bilthoven, The Netherlands (*Brucella *isolated from humans and reference strains). The human isolates from the RIVM were all, except two, isolated from patients in The Netherlands between 1969 and 2008. The strains, together with additional information, are shown in Additional file 1: Table S1.

### MLVA analysis

The target DNA for polymerase chain reaction (PCR) assays was extracted by heating bacterial suspensions in sterilized, demineralized water for 90 min at 95°C. The amplification of the different variable-number tandem repeats (VNTR) was performed as previously described [18,19,29-31]. Moreover, as described by Al Dahouk et al., an additional VNTR was added to the initial MLVA-15 [18,19,29,30].

The PCR amplification was performed in 15-μl volumes containing 1U FastStart Taq polymerase (Roche), 1 × PCR Roche reaction buffer (10 mM Tris-HCl, 2.5 mM MgCl_2_, and 50 mM KCl at pH 8.3), 0.2 mM dNTPs (Roche) and 0.3 μM of each flanking primer. Thermal cycling, conducted on a Peltier Thermal Cycler DNA Engine DYAD (MJ Research), was performed as follows: an initial heating at 95°C for 5 min followed by 35 cycles of denaturation at 95°C for 30 sec, annealing at 60°C for 30 sec and extension at 70°C for 60 sec. A final extension was performed at 70°C for 5 min.

Lab-on-a-chip genotyping was used as previously described to analyze the number of tandem repeats in each locus [18]. The amplification products were loaded into a 96-well or 384-well PCR plates that were prepared according to the manufacturer's recommendations (Caliper HT DNA 5 K Kit, Caliper Life Sciences, Hopkinton, USA). Each chip contained 5 active wells: 1 for the DNA marker and 4 for the gel-dye solution. A marker ladder of MW 100, 300, 500, 700, 1, 100, 1, 900, 2, 900, and 4, 900 bp was used for referencing the molecular weight. The number of samples per chip preparation was 400, equivalent to four 96-well plates or one 384-well plate. After gel preparation, the sample plate was loaded into the plate carrier attached to the robot of the Caliper LabChip 90 (Caliper Life Sciences). During the separation of the fragments, the samples were analyzed sequentially, and electropherograms, virtual gel images and tabulated data were shown. The amplification product size estimates were obtained using the LabChip GX (Caliper Life Sciences) [18]. For each fragment size, the corresponding allele was assigned using the conversion table that was previously described [18]. The assigned number of each tandem repeat was imported into the BioNumerics software package (version 5.10, Applied Maths, Belgium). A clustering analysis was performed using the unweighted pair-group method using arithmetic averages (UPGMA). The UPGMA method assumes a constant rate of evolution, which is presumed for *Brucella *species because genetic recombination in *Brucellae *and horizontal gene transfer among *Brucella *species is low [32,33]. In all of the loci, the differences in the number of repeats were weighted equally because at one locus, multiple tandem repeats can be incorporated during one recombination event.

The publicly available MLVA database for *Brucella *(MLVA-NET for Brucella, http://mlva.u-psud.fr/brucella/) was used to identify or confirm the identity of all of the isolates used in this study. The comparison between the caliper data and MLVA bank showed some discrepancies for the allelic sequences that were obtained using different electrophoretic techniques. Due to the different nature of the gel matrix, these differences were resolved by sequencing [18,30].

### Culture conditions and sample preparation for MALDI-TOF-MS analysis

From a frozen stock, the bacteria were cultured on blood agar plates for at least 48 h at 35°C in the presence of 5% CO_2_. Before sample preparation, the isolates were re-grown for 48 h at 35°C in the presence of 5% CO_2_. Sample preparation was performed according to the company guidelines (Bruker Daltonics, Bremen, Germany). Briefly, 30 colonies were suspended in 300 μl of water (MilliQ, Millipore, Billerica, MA, U.S.) and mixed carefully. Next, 900 μl of absolute ethanol (Fisher Scientific, Loughborough, UK) was added and the suspension was mixed. Subsequently, the suspension was incubated for 90 min to inactivate all of the bacteria. After this inactivation step, the suspension samples were centrifuged for 10 min at 10, 000 g. The supernatant was removed. To remove the remaining ethanol residue, the spinning step was repeated, and the remaining supernatant was removed. Subsequently, 50 μl of 70% formic acid was added to the pellet, and the pellet was mixed. Next, 50 μl of pure acetonitrile (LC-MS grade, Fluka/Aldrich, Stenheim, Germany) was added, and the suspension was mixed carefully. The particulate matter that could not be dissolved was spun down by centrifugation for 2 min at 10, 000 g. Finally, four spots were created, using 0.5 μl of the supernatant per spot, onto a MALDI-TOF target plate (MTP 384 target polished steel #209519, Bruker Daltonics) and air dried. Subsequently, the spots were overlaid with 0.5 μl of α-cyano-4-hydroxycinnamic acid (HCCA, Bruker Daltonics) and a 10 mg/ml acetonitrile/water solution (1:1) with 2.5% trifluoroacetic acid (TFA) (Fluka/Aldrich, Stenheim, Germany) and dried at room temperature.

### Mass spectra acquisition

All of the mass spectra were automatically acquired on a Bruker Autoflex III smartbeam instrument (Bruker Daltonics GmbH, Bremen, Germany) in linear mode using the following parameters: 40% laser intensity, positive polarity, 350 ns PIE delay, 20 kV source voltage 1, 18.7 kV source voltage 2, 8 kV lens voltage, 1.522 kV linear detector voltage, and 800 Da detector gating. Composite mass spectra were generated from 10 different positions per spot using, in total, 2, 000 laser shots at each spot generated by a 200-Hz smartbeam laser (355 nm). The mass spectra were recorded at a mass/charge range between 800 Da and 20 kDa. The instrument was externally calibrated with a bacterial test standard (BTS, Bruker). Furthermore, by including *E. coli *DH5α during each extraction procedure, the complete procedure was validated. For the construction of the custom *Brucella *reference library, 24 MS spectra for each bacterium were generated (eight MS-spectra were generated per day on three different days).

### MALDI-TOF-MS data analyses

The initial data analysis was performed with Bruker Daltonics MALDI Biotyper 2.0 software (Bruker). The raw spectra were automatically pre-processed in a 5-step approach: (1) mass adjustment, (2) smoothing, (3) baseline subtraction, (4) normalization, and (5) peak detection (Bruker). The MLVA genotyping results were used to set up a reference library for *Brucella *species. From each MLVA-cluster except cluster 8, one isolate was selected to generate a custom reference library for the identification of *Brucella *species (Table 1). For cluster 8, two isolates were selected because this cluster contained both *B. suis *and *B. canis *isolates. These isolates, 18 in total, were used to generate the *Brucella *reference library. From each selected isolate, a main spectra (MSP, a 'reference peak list' that is created using a fully automated process in Biotyper 2.0) was created using 24 MS spectra (from three independent measurements at eight different spots) according to company guidelines, using default settings (Bruker). A custom taxonomic tree was created based on the topology of the MLVA tree (Table 1). Subsequently, the MSPs were added to the corresponding taxon nodes. Next, from the remaining 152 isolates, four MS spectra were compared against the generated custom *Brucella *reference library, and the logarithmic score values were calculated. The logarithmic score value is determined by calculating the proportion of matching peaks and peak intensities between the test spectrum and the reference spectra of the database. The highest logarithmic score value is the closest match to a representative isolate in the reference library used. The logarithmic score values range from 0 to 3. If the highest logarithmic score value is < 1.700, the spectrum will be reported as 'not reliable identification', indicating that the spectrum could not be used to identify the strain with the reference library used. A logarithmic score value from 1.700 to 1.999 will be reported as 'probable genus identification', indicating that the genus identification is reliable. Next, a high logarithmic score value from 2.000 to 2.299 will be reported as 'secure genus identification, probable species identification', indicating that the genus identification is secure but that the species identification may be incorrect. A logarithmic score value of 2.300 to 3.000 will be reported as 'highly probable species identification', indicating that the isolate is identified at the species level with a high probability [27].

**Table 1:** Included strains and the taxonomic structure of the *Brucella *library generated using the Biotyper 2.0 program

Genus	Group	Sub-group	MLVA cluster	Strain	Species
Brucella	melitensis/abortus	melitensis	1	Ether	*Brucella melitensis*
			2	16M	*Brucella melitensis*
			3	63/9	*Brucella melitensis*
		abortus	4	98/3033	*Brucella abortus*
			5	W99	*Brucella abortus*/*melitensis*
			6	B19	*Brucella abortus*
			7	Tulya	*Brucella abortus*
	non-melitensis/abortus	suis/canis/ovis	8	RM6/66	*Brucella canis*
			8	686	*Brucella suis *biovar 3
			9	S2 Chine	*Brucella suis *biovar 1
			10	Thomsen	*Brucella suis *biovar 2
			11	Réo 198	*Brucella ovis*
		ceti/pinni/neo	12	09-00388	*Brucella pinnipedialis*
			13	17g-1	*Brucella pinnipedialis*
			14	M78/05/2	*Brucella ceti*
			15	513	*Brucella suis *biovar 5
			16	M 644/93/1	*Brucella ceti*
			17	5K33	*Brucella neotomae*

Apart from the Bruker Daltonics MALDI Biotyper 2.0 data analysis, for presentation purposes, the spectra were converted to the Matlab format. This conversion was performed in two steps: the spectra were first converted into the MZXML format, using the Bruker supplied executable CompassXport.exe, and subsequently to the Matlab binary format using the Matlab routine mzxmlread.m (Matlab 7.5). The spectra presented here were processed further using the Matlab Bioinformatics toolbox (Version 3.0) routines msresample.m for resampling, mslowess.m for smoothing, msbackadj.m for baseline subtraction and finally msnorm.m for normalization of the spectra.

## Results

### MLVA

The MLVA was used to ascertain the identity of all of the isolates used in this study by comparing their MLVA profiles against the publicly available MLVA database for *Brucella *(MLVA-NET for *Brucella*, http://mlva.u-psud.fr/brucella/). All of the isolates except strain W99 were identified at the species level. Strain W99 matched as closely to a *B. abortus *as to a *B. melitensis *in the database, indicating the close relationship between the two species. This isolate is known in the literature as *B. abortus *W99, an A-epitope dominant strain used in a study in which the smooth lipopolysaccharides have been characterized [34]. This W99 strain differs at seven different loci from known *B. melitensis *and *B. abortus *isolates and thus is most likely an outlier.

**Figure 1:**
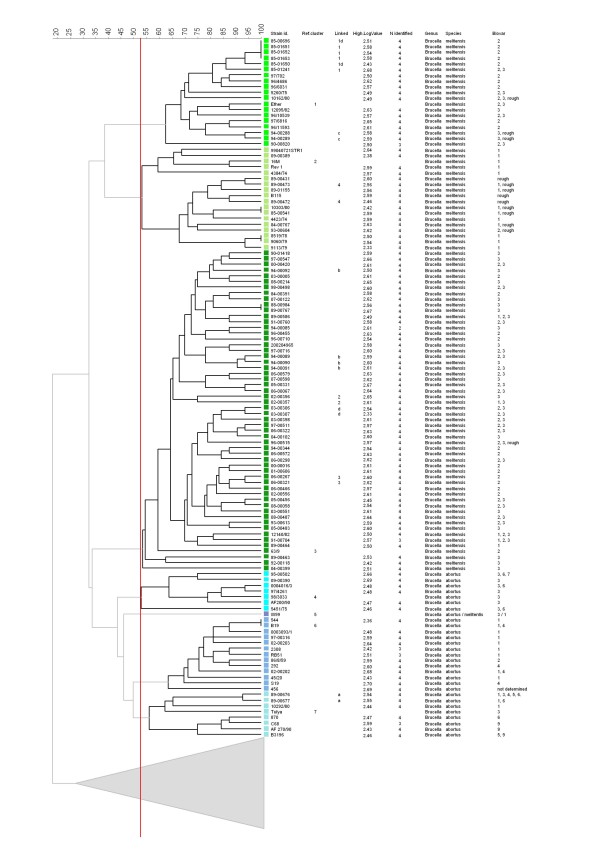
**Partial dendrogram MLVA-16 clustering analysis of 170 *Brucella *isolates, with all 93 of the *B. melitensis *and 29 *B. abortus *isolates included in this study**. The columns present the following data: original strain number [Strain id.], MLVA cluster number reference [Ref. cluster], epidemiologic relatedness (a-d indicate isolates from the same patient, 1-3 indicate isolates that are epidemiologically linked to each other)[Linked], highest logarithmic value of the four generated MS spectra [High LogValue], number of the 4 generated MS spectra corresponding with species identification using MLVA [N identified], genus [Genus], species [Species], and biovar [Biovar] identification based on the MLVA database. The similarity axis is presented in the top left corner. Each color reflects a different cluster with > 52.5% similarity. The group of *'*melitensis-abortus' isolates clustered as follows: *B. melitensis *isolates grouped in Clusters 1, 2, and 3. *B. abortus *isolates grouped in Clusters 4, 6, and 7. Outlier *B. abortus*/*melitensis *W99 is a singleton (Cluster 5).

**Figure 2:**
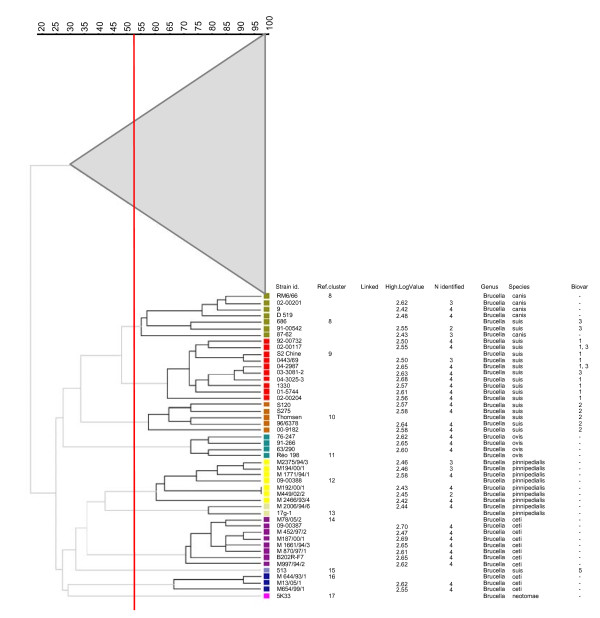
**Partial dendrogram MLVA-16 clustering analysis of 170 *Brucella *isolates, including the 48 isolates from *Brucella *species that were not *B. melitensis *or *B. abortus *included in this study**. The columns present data as described in Figure 1. The similarity axis is presented in the top left corner. Each color reflects a different cluster with > 52.5% similarity. The group of 'non-melitensis/abortus' isolates clustered as follows: Cluster 8 with *B. suis *biovar 3 and *B. canis*; Cluster 9 with *B. suis *biovar 1; Cluster 10 with *B. suis *biovar 2; and Cluster 11 with *B. ovis *isolates. The *'B. ceti/pinnipedialis*/*neotomae' *subgroup clustered as follows: Clusters 12 and 13 with *B. pinnipedialis *isolates and Cluster 14 and 16 with *B. ceti *isolates. Furthermore, this subgroup also contained two clusters with only one isolate (singletons): Cluster 15 with a *B. suis *biovar 5 and Cluster 16 with a *B. neotomae *isolate.

The clustering of the MLVA results using the UPGMA clustering algorithm divided the 170 isolates into 14 clusters and 3 singletons with a genetic similarity of > 52.5% (Figures 1 and 2). The genetic relatedness of > 52.5% was somewhat arbitrarily selected based on the discriminatory power between species and/or biovars. In the dendrogram including the reference strains (Figures 1 and 2), all of the isolates clustered as expected from the literature and the species identification using MLVA (17). Although all of the reference strains were identified as the correct biovar, except for the identification of *B. abortus *biovar 5, which was identified as biovar 5 or 9, identification to the biovar level using MLVA proved to be ambiguous because sometimes the profiles were found to be equally similar to multiple biovars. Thus, the biovar could not be assigned to 8 (29%), 28 (30%), and 2 (11%) of the *B. abortus*, *B. melitensis*, and *B. suis *isolates, respectively. Cluster 10 only contained isolates of *B. suis *biovar 2. However, the other clusters contained multiple biovars. Based on genetic similarity, these clusters and the singletons could be divided into two genetically related groups. The first group, *B. melitensis*/*abortus *(BAM), consists of 6 clusters and 1 singleton (W99) isolate, which are all *B. melitensis *or *B. abortus *species. The second, non-BAM group is genetically more diverse and contains 8 clusters and 2 singletons comprising the other *Brucella *species (*B. suis*, *B. canis*, *B. ovis*, *B. pinnipedialis*, *B. ceti*, and *B. neotomae*). *B. suis *biovars 1, 2, and 3 and *B. canis *are genetically highly related, whereas *B. suis *biovar 5 is genetically distinct from other *B. suis *biovars. Epidemiologically related strains, from the same outbreak or isolated from the same patient, were grouped in the same clusters with a genetic relatedness of 70% or more (Figures 1 and 2).

### MALDI-TOF-MS

**Figure 3:**
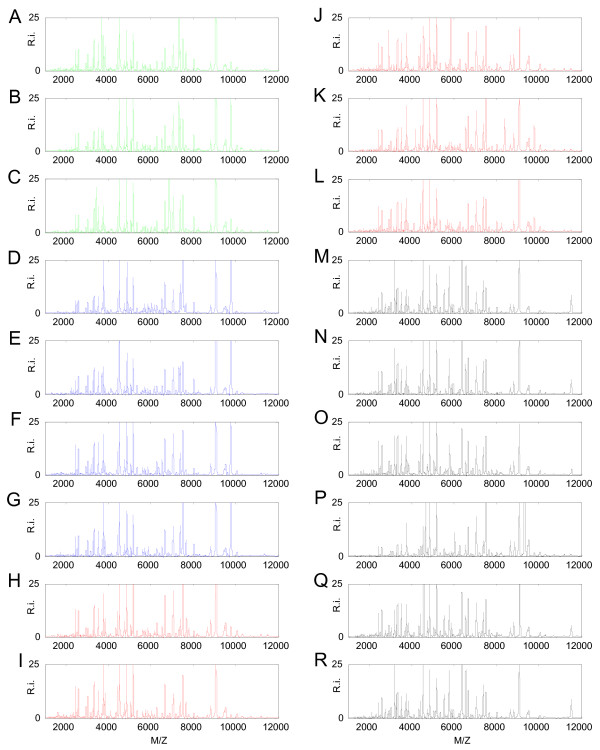
**Representative MALDI-TOF-MS spectra of the *Brucella *strains used as references in the generated *Brucella *reference library in the range of 1, 000 to 12, 000 Da**. The relative intensity (R.i) is shown as a percentage of the total intensity on the y-axis, and the mass to charge ratio (M/Z) is shown on the x-axis. A) *B. melitensis *Ether. B) *B. melitensis *16 M. C) *B. melitensis *63/9. D) *B. abortus *98/3033. E) *B. abortus*/*melitensis *W99. F) *B. abortus *B19. G) *B. abortus *Tulya. H) *B. canis *RM6/66. I) *B. suis *biovar 3 686. J) *B. suis *biovar 1 S2 Chine. K) *B. suis *Thomsen biovar 2. L) *B. ovis *Réo. M) *B. pinnipedialis *09-00388. N) *B. pinnipedialis *17 g-1. O) *B. ceti *M78/05/02. P) *B. suis *biovar 5 513. Q) *B. ceti *M 644/93/1. R) *B. neotomae *5 K33.

The 608 MS spectra derived from 152, mostly clinical, isolates were compared against the reference library generated for *Brucella *species. Representative MS spectra from the 18 isolates selected for the *Brucella *reference library are shown (Figure 3). Minor visual differences (peaks and intensities) among the MS spectra are detectable. A total of 25 MS spectra had a logarithmic score value from 2.000 to 2.299, indicating 'secure genus identification, probable species identification'. The highest logarithmic score values of the remaining 583 MS spectra were between 2.300 and 3.000, which indicate 'highly probable species identification'.

Because *Brucella abortus *W99, a singleton strain, is equally similar to *B. abortus *as to *B. melitensis*, we interpreted this strain as a potential *B. melitensis *strain. When identification at the species level is based on a 'majority rule' (i.e., identification is based on the species indicated by at least three out of four MS spectra), 149 (98%) isolates were correctly identified at the species level. Further, when instead of the majority rule, the identification at the species level was based on the highest of the four logarithmic values, which was always > 2.299, 151 (99.3%) of the isolates were correctly identified at the species level, while only 1 (0.7%) isolate was mistakenly identified as *B. canis *instead of *B. suis*.

The isolates 03-3081-2, 04-2987, and 02-00117, which were identified as *B. suis *biovar 3, 1 or 3 and 1 or 3, respectively, based on their MLVA profile similarity, were all grouped into cluster 9, which only contained *B. suis *biovar 1 isolates. Therefore, these three isolates are most likely *B. suis *biovar 1.

The MLVA data further demonstrated that the *B. suis *biovars 1 (MLVA cluster 9) and 2 (MLVA cluster 10) are genetically distinct clusters, whereas *B. suis *biovar 3 grouped together with *B. canis *isolates in a single genetic cluster (MLVA cluster 8). Next, we determined if the *B. suis *biovars could be identified to their biovar level using MALDI-TOF-MS. Of the 4 *B. canis *isolates and 14 *B. suis *isolates (9 were *B. suis *biovar 1, assuming that the isolates 03-3081-2, 04-2987, and 02-00117 were biovar 1 as discussed previously, 4 were *B. suis *biovar 2, and 1 was *B. suis *biovar 3), only the *B. suis *biovar 3 isolate was mistakenly identified as *B. canis *using either the 'majority' or 'highest score' rule. For these results, we have considered the library strain W99 to be *B. melitensis*. Removing W99 from the *Brucella *reference library and comparing the 604 MS-spectra against this library only slightly influenced the classification results.

## Discussion

An immediate response is required to mitigate the effects of a biological attack. The timely detection of a biological event is essential to respond. Then, exposure to the agent may be reduced by the application of protective measures, the most important of which is airway protection. *B. melitensis*, *B. suis*, and possibly *B. abortus *are considered to be potential warfare agents. To date, the detection and identification of *Brucella *species is laborious and time consuming. However, MALDI-TOF-MS may provide a new and rapid method that enables the quick identification of microorganisms. *Brucella *species are very difficult to identify. Not only are the species genetically highly related but also the taxonomy of *Brucella *species is open to debate because discrepancies in the nomenclature used were observed in the past [33]. First, *B. suis *is paraphyletic, from a genetic point of view because it contains not only *B. suis *but also *B. canis *[32]. Further, whole-genome sequencing demonstrated that *B. canis *is genetically highly similar to *B. suis *biovars 3 and 4 [32]. Likely, *B. canis *has arisen from its ancestor *B. suis*. In contrast, *B. suis *biovar 5 is genetically much more related to *B. pinnipedialis *and *B. ceti *than to the other *B. suis *biovars [19,32]. Second, Maquart and coworkers showed that *B. ceti *is divided into two separate clusters, one cluster of which was genetically more related to *B. pinnipedialis *than to the other cluster of *B. ceti *[20]. Third, *B. melitensis *from the western Mediterranean is genetically closer to *B. abortus *than to *B. melitensis *of eastern Mediterranean or American origin [20]. Clearly, the taxonomy of *Brucella *species is based on pathogenesis, host specificity, and geographic source rather than on genetic relationships. These issues complicate the development of new identification methods but also complicate the interpretation of the identification results, which is illustrated by the fact that no specific biological markers for *B. suis *have been identified [14,33]. A new classification, based on genetics, of the taxa within the genus *Brucella *is needed, rather than assigning the names of the conventional species and biotypes to the taxa created using molecular methods. Only species and biotypes that are both genotypically and phenotypically coherent can be maintained. Because this reclassification is beyond the scope of this article, the identification of the *Brucellae *used in this study was based on the MLVA database.

The previously developed 16-MLVA method has been shown to have a high discriminatory power and is able to correctly identify all of the known species of the *Brucella *genus [13,18-20]. Therefore, identification at the species level of isolates based on comparisons with the MLVA database should be considered reliable. However, identification at the biovar level using MLVA analysis proved to be ambiguous, especially for *B. melitensis *and *B. abortus*, as described previously (1, 14). Although we found some discrepancies in the MLVA profiles of the reference strains between the publically available database and our results, these differences are likely due to difficulties in the interpretation of the MLVA profiles because of the small and contiguous sizes of some alleles (Bruce 08, 21, 16 and 19).

In this study, we demonstrated that MALDI-TOF-MS enables the identification of *Brucella *isolates at the species level. Predominantly, isolates of *B. melitensis *and *B. abortus*, the main cause of human brucellosis in The Netherlands, were tested, and all of the isolates were identified correctly. Although the number of *B. suis *biovar 1 and 2 isolates in this study was limited, the isolates present were correctly identified at their biovar level as well. The interpretation of the one isolate of *B. suis *biovar 3 as *B. canis *is likely due to the high similarity of *B. suis *biovars 3 and 4 to *B. canis *[32]. A previous study by Ferreira et al. could not discriminate at the species level [25]. The constructed reference library by Ferreira et al. did not represent the complete diversity between *Brucella *species, which could possibly explain the reduced discriminatory power to the species level. Furthermore, we noticed that strain NCTC 10098 was a *B. melitensis *according the NCTC and not a *B. suis *as it has been used by Ferreira et al. [25]. In addition, in the library of Ferreira et al., no *B. abortus *isolates of cluster 4 (Figure 1) were included.

This study presents an additional observation that further highlights the controversy of combining molecular data with the conventional taxonomy of the genus *Brucella*. As mentioned earlier, the results described are based on the assumption that the *B. abortus *strain W99 is phenotypically more strongly related to *B. melitensis *than to *B. abortus*. This assumption was supported by the results because the MS spectra of the 80 isolates that were identified to be *B. melitensis *using MLVA closely resembled the MS spectrum of W99, whereas none of the MS spectra derived from *B. abortus *isolates had a similar resemblance. Thus, phenotypically, strain W99 is more closely related to *B. melitensis *than to *B. abortus*. It is possible that strain W99 is related to the common ancestor of the BAM group.

MALDI-TOF-MS identification is based on profiles derived from the proteome. To counter the inherent minor variations found between measurements of the MS spectra, the MS profiles in the reference library constructed here consist of the mean of 24 MS spectra. The fact that the identification of genetically highly related species appeared to be feasible demonstrates that even minor genetic differences are translated to specific proteomic differences.

## Conclusions

Discrepancies between classical taxonomy and the genetic relatedness of species and biovars complicate the development of detection and identification assays. Despite these difficulties, the accurate identification of *Brucella *species was achieved with MALDI-TOF-MS by constructing a *Brucella *reference library based on genetic relationships according to MLVA data. We conclude that MALDI-TOF-MS can be developed into a fast and reliable identification method for genetically highly related species when potential taxonomic and genetic inconsistencies are considered during the generation of the reference library.

## Authors' contributions

FL participated in the design of the study and coordinated the MLVA work; FR participated in the design of the study and critically revised the manuscript; RDS executed the MLVA experiments, analyzed the data and drafted the manuscript; RP participated in the analysis of the MALDI-TOF-MS data; AdJ executed the MALDI-TOF-MS experiments and participated in the MALDI-TOF-MS data analysis; JK participated in the design of the study; AvdL executed the MALDI-TOF-MS experiments; IVV executed the MALDI-TOF-MS experiments; SF executed the MLVA experiments; HJJ participated in the design of the study and critically revised the manuscript; JVdP participated in the design of the study and critically revised the manuscript; and AP participated in the design of the study, performed data analysis on the MLVA and MALDI-TOF-MS data, coordinated the MALDI-TOF-MS experiments, and drafted the manuscript. All authors read and approved the final manuscript.

## Supplementary Material

Additional file 1 **Table S1**. Strains used during the study with additional information. Click here for file
